# Relationship between Painful Crisis and Serum Zinc Level in Children with Sickle Cell Anaemia

**DOI:** 10.1155/2011/698586

**Published:** 2010-11-25

**Authors:** Edamisan Olusoji Temiye, Edem Samuel Duke, Mbang Adeyemi Owolabi, James Kweku Renner

**Affiliations:** ^1^Department of Paediatrics, College of Medicine, University of Lagos (CMUL), P.M.B 12003, Lagos, Nigeria; ^2^Critical Rescue International (CRI), Plot 144, Oba Akran Road, Ikeja, Lagos, Nigeria; ^3^Department of Pharmaceutical Chemistry, Faculty of Pharmacy, College of Medicine, Idi-Araba, University of Lagos, P.M.B. 12003, Lagos, Nigeria

## Abstract

Sickle cell anaemia (SCA) is associated with zinc deficiency; zinc supplementation may ameliorate some of its clinical manifestations including the relief of painful crisis. *Subjects and Methods*. Serum zinc levels were determined in 71 children with SCA and painful crisis and in equal numbers in steady state. Seventy-one children with AA genotype acted as controls. Qualitative assessment of zinc content of 24-hour dietary recall and the last meal consumed before blood was drawn was taken. Serum zinc was determined using atomic absorption spectrophotometer. Haemoglobin concentration and packed cell volume (PCV) were determined using standard methods. *Results*. The mean serum zinc concentration in the study was less than international reference range. The controls had significantly higher serum zinc concentrations than the SCA group (42.7 ± 13.6 versus 32.3 ± 14.0 *μ*g/dL, *P* < .000); this difference was due to the significantly lower values of serum zinc in SCA with painful crisis compared with the remaining two groups *F* = 30.9, *P*<.000. There was a positive correlation between serum zinc and haemoglobin concentration only in the control group (*r* = 0.4; *P* = .001). *Conclusion*. The serum zinc levels in this study were low. Painful crisis in SCA may exert greater demand for zinc utilization in children with SCA thereby resulting in lower serum levels.

## 1. Introduction

Sickle cell anaemia is the most common inherited disorder of the black race. It affects red blood cells resulting in chronic haemolytic anaemia of varying severity and in some patients, periodic painful crisis caused by the occlusion of small blood vessels by spontaneous intravascular sickling with multiorgan affectation [1, 2].

Brewer and Oelshlegel discovered that calcium binding to red cell membrane is responsible for the formation of irreversible sickled cells and that the antisickling effect of zinc in sickle cell anaemia is due to its ability to antagonize calcium binding to red cell membrane [3]. In sickle cell anaemia, there is also increased oxidative stress and peroxidation as well as low antioxidant potential which predisposes the patients to vaso-occlusive crisis [4–6]. Zinc exerts its antioxidant action by inhibition of lipid peroxidation which occurs in red blood cells and liver thereby stabilizing biomembranes and biostructures thus protecting the body against oxidative stress [5, 6]. These effects of zinc are believed to give zinc its ability to reduce vaso-occlusive crisis in sickle cell anaemia. 

Various studies have shown that zinc deficiency may be common in SCA patients. This has been attributed to chronic haemolysis that occurs in these patients, increased demand and utilization, along with the secondary loss of zinc in the urine [1, 7, 8]. Furthermore, supplementation of zinc in sickle cell anaemia has been reported to improve wound healing, decrease incidence of infection, improve the age of attaining secondary sexual characteristics, reverse dark adaptation of the eyes, and accelerate growth [8–12].

Although it has been suggested that zinc therapy could reduce painful crisis [13, 14]; however, it has not been shown with certainty that zinc deficiency in sickle cell anaemia is directly related to the development or severity of vaso-occlusive crisis. Prasad et al. [15], in 1976 administered oral zinc sulfate to 10 adults who had severe painful crisis and found symptomatic improvement in 8 of the subjects, which they attributed to the antisickling effect of zinc. The significance of their findings may be limited because of the very small sample size and lack of control subjects in the study. In a placebo-controlled double-blind study, 145 sickle cell adult subjects were treated with either 220 mg of zinc sulphate 3 times daily or placebo [14]. After 18 months, the zinc-treated subjects had an average of 2.5 crises, compared to 5.3 in the placebo group; but the severity of painful crisis was not reduced. A study in Ibadan, Nigeria [16] found no relationship between serum zinc level and the different degrees of clinical severity of the disease in these subjects. They observed that the level of zinc in each patient, though significantly lower than the controls, had no correlation with the haematocrit, frequency of bone pain crisis, and susceptibility to infection. The sample size in their study was also small making it difficult to draw any valid inference.

It is probable that zinc deficiency may be associated with painful crisis. However, these authors are not aware of any previous work comparing zinc status in sickle cell anaemic patients in vaso-occlusive crisis with those in steady state. This study is therefore designed to assess the level of serum zinc in sickle cell anaemia children in painful state (vaso-occlusive crisis) as compared with those in steady state. We also relate the levels of serum zinc with diet and haemoglobin levels in the subjects.

## 2. Subjects and Methods

This was a case-controlled study of patients at the Lagos University Teaching Hospital (LUTH), Nigeria. The sample size for the study and the number of subjects for each arm were determined using standard formulae for population study [17], using the prevalence rate of SCA in western Nigeria [18] and the rate of deficient erythrocyte zinc level in sickle cell anaemia children in Zaria [19]. One hundred and forty-two paediatric subjects aged between one and 12 yrs who had haemoglobin genotype SS were studied. Of these, 71 were in had bone pain crisis and were recruited consecutively from both the haematology clinic and Children's Emergency Room (CHER) the remainder were in steady state recruited from the sickle cell clinic. The control group consisted of 71 apparently well subjects whose haemoglobin genotype was AA. They were recruited from the Well Baby and Children's Outpatient Clinic. Both the SCA in steady state subjects and the controls were selected such that they were matched for age and sex with the subjects who had painful crisis. 

In this study, sickle cell anaemia in painful crisis was defined as subjects who had pains at the time of recruitment or within 48 hours before recruitment in any of the limbs [20], while steady state was defined as subjects who were apparently well without evidence of recent infection, bone pain, or other problems for at least 4 weeks before recruitment [21]. The study, which was approved by the research and ethics committee of the hospital, lasted for a period of 12 months. Other inclusion criteria were that study population were not on any zinc-containing medications, they were fed at least 4 hours before blood collection, and had not been transfused with blood or blood products in the previous three months preceding study. Also included were subjects who did not have any symptoms or signs of infection such as fever (axillary temp < 37.0°C), acute respiratory infection, diarrhoea, or malaria. Additionally, controls had no evidence of chronic disease including protein energy malnutrition. The haemoglobin genotype of all the subjects was determined by haemoglobin cellulose electrophoresis. 

After informed consent, a detailed history of each child from the care-giver was taken; these included the mother's and father's educational status and occupation, 24-hour meal recall, date of last blood transfusion, and the presence or absence of pain in the limbs. Physical examination including assessment for the presence or absence of fever (temp > 37.0°C), jaundice, pallor, finger clubbing, liver size, spleen size, and tenderness in the limbs, the weight, and length/height was done. Previous health status of each subject was obtained by examining the hospital records. Five mL of blood was collected from each subject by venepuncture after appropriate skin preparations with a 21-gauge stainless-steel needle with a polypropylene syringe (Norm-Ject; Henke Sass Wolf GMBH, Germany) [22]. Two mL of blood was transferred into a sodium ethylenediamine-tetra-acetate (EDTA) bottle for haemoglobin (Hb), Packed cell volume (PCV), and haemoglobin genotype determination using standard laboratory procedure. The remaining 3 mL was transferred into zinc free nonheparinized bottles previously washed clean of possible zinc contamination by leaving them soaked in 10% nitric acid for 24 hours and rinsed three times with deionised water. The clotted blood sample in the zinc-free bottles was centrifuged at 1500 rpm for 10 minutes and the serum removed with zinc free Pasteur Pipette prepared by previously washing with 10% nitric acid and thoroughly rinsed with deionised water as described for the bottles above [23]. The serum samples were then stored at −20°C pending analysis [24]. All haemolysed samples were discarded. Before the analysis, the serum samples were allowed to come to room temperature and each mixed by gently inverting the tube four times. Serum zinc level was determined using an atomic absorption spectrophotometer (PU9100X, Philips, Holland) at the Food Technology laboratory of the Federal Institute of Industrial Research Oshodi (FIIRO) Lagos, Nigeria and following the method of Smith et al. [22]. The concentration of each sample, which was expressed in parts per million, was extrapolated from the calibration curve prepared from the standard zinc calibration curve and converted to *μ*g/dL by calculation.

## 3. Classification of Zinc Content in 24-Hour Meal Recall

The zinc contents of 24-hour meal recall and the last meal consumed before blood was taken were classified based on the concentration of zinc in food items consumed by the subject. Thus each food item was classified as containing high, moderate, or low zinc [25, 26]. Meals which contain items such as plantain, red beef, sea foods, egg, milk, cocoa products, and fish were classified as high zinc content, while beans, cereals made out of maize and rice products contain moderate zinc. Cassava products were classified as having traces or low zinc content [25, 26]. For the purpose of this study, a subject who had more than 2 high zinc containing items with any 3 moderate zinc containing items or all high zinc containing items was classified as having high zinc diet. A subject who had one high zinc containing item and less than 3 moderate zinc containing items or had all moderate zinc containing items was classified as having moderate zinc, while a subject who had one moderate zinc or one high zinc containing item with low zinc containing item, or only low zinc containing items, was classified as having low zinc.

## 4. Data Analysis

Data generated was analysed using the SPSS statistical package version 11. Frequency distributions were generated for all categorical variables. The measures of location were determined for quantitative outcome such as age, zinc level, and haematocrit. Statistical significance between two means was assessed using Student *t*-test, while one-way analysis of variance was used where there were multiple means. Where there was significant difference within means, post hoc test was applied to determine where the level of significance occurred. Chi-square (*χ* ^2^) test was applied for categorical variables and correlation analysis as applicable. Differences between values were accepted as statistically significant where probability was less than .05.

## 5. Results

Two hundred and thirteen children aged between one and 12 yrs were recruited into the study; seventy-one were controls with haemoglobin genotype AA, while one hundred and forty-two subjects had haemoglobin genotype SS. Seventy one of the subjects with sickle cell anaemia were in steady state while the rest had vaso-occlusive bone pain crisis. Eighty-two of the study subjects were males and 60 were females giving a male : female ratio of 1.3 : 1. This difference was not statistically significant. Also, the male: female ratio for each of the subgroup was not significantly different. 

The mean ages of the subjects in control group and those with sickle cell anaemia were 71.3 ± 34.3 months and 73.6 ± 31.4 months, respectively. The difference was not statistically significant. (*t* = 0.5, *P* = .64). Although the control subjects were heavier than the SCA subjects (weight (kg), 21.1 ± 8.0 versus 19.1 ± 6.0, *t* = 2.1, *P* = .04) and they were also taller, (height (cm) 115.7 ± 21.1 versus 112.1 ± 17.6, *t* = 1.3, *P* = .2), the differences were not statistically significant. However, the control subjects had a mean packed cell volume of 33.1 ± 5.6% and haemoglobin of 11.3 ± 2.0 g/dL which were significantly higher (*t* = 16.9, *P* = .000) than the SCA group with a mean packed cell volume of 21.6 ± 4.2% and haemoglobin concentration of 7.4 ± 1.4 g/dL, respectively. The mean serum zinc concentration in the control subjects was also significantly higher than the sickle cell anaemia group (42.7 ± 13.6 versus 32.3 ± 14.0 *μ*g/dL, *t* = 5.2, *P* = .000)

When the sickle cell anaemia group was subdivided into those in steady state and those with bone pain crisis, the mean age remained not significantly different although the mean age for subjects with painful crisis was higher than those of sickle cell anaemia in steady state and the control subjects. Sickle cell anaemia subjects in steady state were however significantly shorter than those with painful crisis and with haemoglobin AA. Similarly, sickle cell anaemia subjects in steady state were significantly lighter than the controls (*P* = .01) subjects and sickle cell anaemia subjects with painful crisis. The significant difference observed in the haemoglobin concentration and packed cell volume was found to be between the control group and the sickle cell anaemia in painful crisis and sickle cell anaemia in steady state. Although the sickle cell anaemia in painful crisis had higher haematological parameters than the steady state subjects, the difference was not statistically significant. The mean serum zinc concentration in subjects with haemoglobin genotype SS in steady state of 38.4 ± 13.8 *μ*g/dL was significantly higher than in subjects with bone pain crisis of 26.3 ± 11.3 *μ*g/dL (*P* < .001). The result also showed significantly higher mean serum zinc concentration levels (*P* < .001) in controls when compared with subjects in bone pain crisis. However, the differences between control and steady state subjects were not statistically significant, Table 1.

The subjects were arranged into four age groups. Thirty three subjects (15.5%) were between 13 and 35 months of age, 57 (26.7%) were in the age group of 36–71 months, the majority (83, 39.0%) were between 72 and 107 months of age and the remaining 40 (18.8%) were between 108–144 months of age. There were, however, no significant differences in the numbers of subjects in the different age groups (*P* > .03). The mean serum levels of zinc for the age groups for all the subjects ranged from 36.21 ± 12.8 *μ*g/dL for age group of 13–35 months, 39.5 ± 15.6 *μ*g/dL for age group of 36–71 months, 35.6 ± 16.0 *μ*g/dL, to 30.7 ± 10.2 *μ*g/dL, for age groups of 108–144 months. The differences in the values were statistically significant *F* = 2.9, *P* = .04.

**Figure 1:**
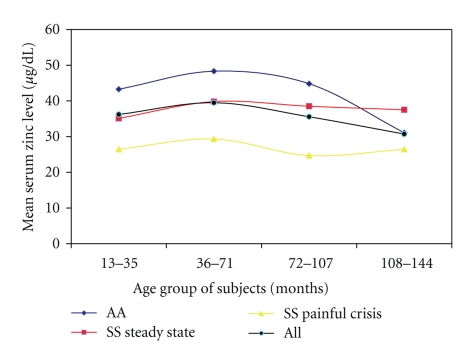
Mean zinc concentration by age groups in the three subgroups.

When the subjects were grouped into controls, sickle cell steady state, and painful crisis, however, the control subjects had higher mean serum zinc levels than the sickle cell anaemia subgroups in all age groups except in the age group of 108–144 months where the steady state subjects had higher levels than the control group. These differences were statistically significant and the level of significance was between the controls and the sickle cell anaemia subjects in painful crisis except in the age group of 108–144 months where the level of significance was between the sickle cell anaemia in steady state and SCA in painful crisis. Among the controls, there was a significant fall in serum zinc levels as the subjects get older, *F* = 5.0, *P* = .004, but such decrease in zinc levels with age was not observed among the sickle cell subgroups; *F* = 0.8, *P* = .3 (in sickle cell anaemia in steady state and *F* = 0.5, *P* = .7 in sickle cell anaemia subjects in painful crisis). In all the age groups, the SCA in painful crisis had serum zinc levels significantly lower than those of controls and subject with SCA in steady state (Figure 1).

**Figure 2:**
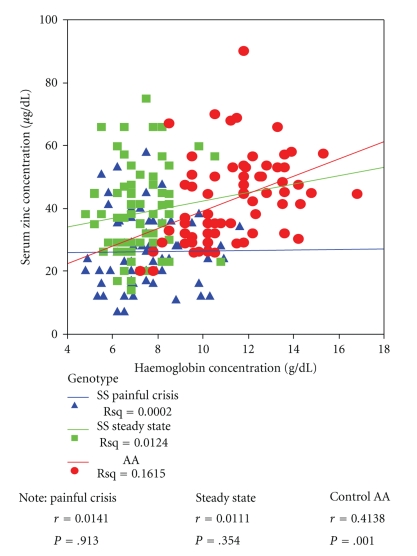
Linear relationship between haemoglobin concentration to serum zinc levels in Control, SCA in steady state, and SCA in painful crisis.

Figure 2 shows the correlation between haemoglobin and serum zinc concentrations. There is a positive correlation between haemoglobin concentration and zinc levels in the control group and this was significant (*r* = 0.4, *P* < .001). There was no correlation observed in the steady states subjects (*r* = 0.01, *P* = .4) and in subjects in bone pain crisis (*r* = 0.01, *P* = .9).

The effect of dietary intake of zinc-containing meals on serum zinc was assessed. Ten (4.7%) subjects were assessed to have consumed meals low in zinc content in the last 24 hours, 6 of these were SCA in steady state and 3 were SCA in painful crisis; the remaining subject in this category was the control. Ninety seven (45.5%) subjects, consisting of 18 controls, 31 SCA in steady state, and 48 SCA in painful crisis had food items with moderate zinc content. The remaining 106 (49.8%) subjects of whom 52 were in control group, 34 SCA in steady state, and 20 SCA with painful crisis had food items containing high level of zinc.

When the zinc content of food items consumed by subjects 24 hour prior to collection of blood for analysis were compared with mean serum zinc concentration, it was found that subjects in the control group whose meal were adjudged to contain moderate amount of zinc had mean serum zinc concentration of 41.1 ± 17.2 *μ*g/dl compared to 43.3 ± 12.5 *μ*g/dl in those whose food items contained high amount of zinc. This difference was however not statistically significant. In the SCA group, subjects in the steady state who consumed low zinc items in their meal had serum zinc concentration of 30.7 ± 12.1 *μ*g/dL, those that consumed moderate zinc levels had 40.8 ± 14.0 while those that consumed high zinc levels had a mean value of 37.6 ± 13.7. This difference was not statistically significant. SCA subjects with painful crisis who consumed low zinc containing meals had mean serum concentration of 33.9 ± 20.7 *μ*g/dL. For those who consumed moderate zinc containing items had mean serum zinc level of 24.8 ± 10.5 *μ*g/dL, and for those that took high zinc containing items their mean serum zinc concentration was 28.5 ± 11.4 *μ*g/dL. Again this difference was not statistically significant. However, across the subgroups, control subjects had mean serum levels of zinc that were significantly higher than those in the SCA subgroups among subjects that consumed moderate to high zinc containing meals, Table 2.

When the zinc contents of the last meals consumed before blood was taken were considered, most subjects, 164 (77.0%) of whom 47, 59, and 58 were in the control, SCA steady state, and SCA painful crisis subgroups, respectively, consumed meals containing moderate zinc contents. Thirty-five (16.4%) subjects had food items containing high zinc content; 21 of these subjects were in control group while 6 and 8 subjects were in SCA in steady state and painful crisis, respectively. The remaining 14 (6.6%) (3 in control group, 6 SCA in steady state, and 5 in SCA painful crisis) had food items containing low zinc contents.

Using the content of the last meal consumed it was found that, except in the control groups, those that consumed high zinc containing meals had highest mean serum zinc levels. This was significant among the SCA in steady state, *F* = 7.0, *P* = .002, and just reached significant level in SCA in painful crisis, *F* = 3.1, *P* = .05. Within the subgroups, those with low zinc consumption had no significant difference, the mean serum zinc level was however significantly lower in SCA in painful crisis than those in controls and steady state who took food items with moderate zinc content. Similar trend was observed in those who consumed food items containing high zinc contents; again the mean serum level for SCA in painful crisis was significantly lower than those of controls and SCA in steady state, Table 3.

## 6. Discussion

The serum zinc levels in this group of Nigerian children were found to be lower than the internationally acceptable normal values of 50–150 *μ*g/dL {7.65–22.95 *μ*mol/L} [27]. This is also true for the control group as well as those with sickle cell anaemia. Several studies have indicated that nutritional zinc deficiency is widespread all over the world. Akinkugbe and Ette [28] observed that low zinc levels are common in Nigerian children. Important risk factors related to zinc deficiency are common use of grain proteins and low content of zinc in breast milk of mothers and weaning food [27, 28]. These factors may be important in our subjects as more than 80% were on food containing low or moderate amount of zinc in their last meal before blood was drawn for analysis. This should reflect more accurately the nature of meal the child is taking than the 24-hour recall. Most of these food items contained high level of cassava- and grain-based diet which limits the quantity of zinc available for absorption due to their high phosphate and phytate content as zinc forms an unavailable complex with these compounds [27–29].

Although the subjects had low serum zinc levels, symptoms attributable solely to zinc deficiency were not found, especially in the control subjects. It is difficult to say at what level of serum zinc that symptoms of deficiency would appear, and the reasons for lack of symptoms in the control subjects is unclear. The significantly lower values of zinc observed in children older than 107 months in the control group probably reflect increased demand for zinc in already suboptimal zinc nutriture in these subjects at the commencement of pubertal growth [30].

In all age groups, the serum zinc levels were significantly lower in the SCA group than in the controls. This finding has been reported in several communications [6, 7, 15, 30] and has been attributed to several factors including the chronic haemolysis that characterizes sickle cell anaemia leading to loss of zinc from red blood cells which is an important storage site for zinc [6, 28]. Furthermore, there is a defective zinc homeostasis as a result of excessive excretion of zinc in urine or abnormal renal tubular reabsorption of zinc due to the sickling phenomena, increased demand and consumption due to increased oxidative stress and sickle cell redox imbalance [6, 8, 10]. The further lowering of serum zinc in SCA subjects with painful crisis in this study was an interesting finding. It could be postulated that SCA subjects who are more severely zinc-deficient would have more painful crisis. On the other hand, frequent painful crisis might interfere with nutrition and lead to more severe nutritional deficiencies [6]. 

Zinc is the most abundant intracellular element with 85% of total body zinc found in muscle and bone; less than 0.1% of total body zinc is in the plasma [31]. Plasma zinc is maintained by continuous shift from the intracellular sources and absorption from the intestine. Hence plasma zinc is a poor indicator of total body zinc. The retention time of zinc in the blood is estimated as 2.3 days [32]; however, metabolic stress, such as infection and acute illnesses which are more common in children with sickle cell disease compared to children with haemoglobin genotype AA, increases intracellular shift of zinc into the liver and lowers plasma level even when total body zinc level is unchanged [33]. As already noted above, the low plasma zinc level in this study in sickle cell anemia subjects with painful crisis may indicate the effects of stress in increasing the intracellular shifting of zinc into the liver from the plasma of these subjects rather than increased loss of zinc from the body.

This study showed a positive correlation between the serum zinc and haemoglobin in the control group. This is in conformity with the findings of Akinkugbe and Ette [28], whose study was on serum zinc and haemoglobin levels in children with common paediatric problems. However, there was no correlation between serum zinc and haemoglobin in the subjects with SCA; this is in conformity with the finding of Akenami et al. [16] in sickle cell anaemia children. The reason for this is not clear. It may be due to the chronic haemolytic state experienced by sickle cell anaemia subjects, leading to loss of zinc at it is storage site (red blood cell) and it is urinary loss. Further studies are required to explain this finding. 

Zinc deficiency is associated with poor growth, among other clinical manifestations [8, 34]. In this study, the control group had significantly higher weight and height than the sickle cell anaemia group in steady state but not with the SCA group with painful crisis. This may be attributed to the growth retardation associated with zinc deficiency. However, one would have expected the SCA children with painful crisis who also had the lowest levels of serum zinc to be the most affected in growth, but this was not the case. The Haemoglobin concentration in the SCA with painful crisis was also higher than those without painful crisis. It is possible that the higher oxygen-carrying capacity of children with painful crisis had a better effect on growth than the level of plasma zinc. Since there was also no significant difference in the quality of the meals consumed in terms of zinc content between these groups, it is possible that the lower serum zinc concentration in SCA subjects with painful crisis resulted from more severe disease with higher oxidative stress leading to higher utilization of zinc rather than a poorer serum zinc status. 

Since this study was a cross-sectional one, it was important to observe whether the zinc status of these subjects improved several days after recovery from their painful episodes or whether zinc therapy would have aborted or shorten periods of painful episodes. These should form the basis for our next study in these children.

## Figures and Tables

**Table 1:** Mean haematological parameters and serum zinc concentration in Cases and Controls.

Parameter	Genotype (Mean ± 2SD)			*F*	*P*-value
	Genotype AA (*n* = 71)	Sickle cell anaemia			
		Steady state *n* = 71	Painful crisis *n* = 71		
Age (mo)	71.3 ± 34.3	67.2 ± 30.1	79.9 ± 31.7	2.9	.06
Weight (Kg)	21.1 ± 8.0	17.9 ± 5.4	20.3 ± 6.3	4.6	.01
Height (cm)	115.7 ± 21.1	108.4 ± 16.8	115.7 ± 17.8	3.6	.03
Hb (g/dL)	11.3 ± 2.0	7.1 ± 1.1	7.6 ± 1.6	144.9	.00
PCV (%)	33.1 ± 5.6	20.9 ± 3.4	22.3 ± 4.8	143.9	.00
Serum Zinc Conc (*μ*g/dL)	42.7 ± 13.6	38.4 ± 13.8	26.3 ± 11.3	30.9	.00

*P* < .05 is significant.

**Table 2:** Relationship between zinc content of 24-hour dietary recall and mean serum zinc level.

Zinc content of meal	Mean serum zinc concentration *μ*g/dL (± SD)			*F*	*P*-value
	Genotype AA	Sickle cell anaemia			
		Steady state	Painful crisis		

Low	45.0^††^	30.7 (12.1)	33.9 (20.7)	*t* = 0.3	.8
Moderate	41.1 (17.2)	40.8 (14.0)	24.8 (10.5)	18.4	.000
High	43.3 (12.5)	37.6 (13.7)	28.5 (11.4)	9.9	.000

^††^Only one subject in the control group had low zinc item and was removed from the statistical analysis; therefore, student *t*-test was used to compare the mean zinc level between sickle cell anaemia subjects in steady state and painful crisis in the first row.

**Table 3:** Relationship between zinc content of last meal consumed before taking blood and mean serum zinc level.

Zinc content of meal	Mean serum zinc concentration *μ*g/dL (± SD)			*F*	*P*-value
	Genotype AA *n* = 71	Sickle cell anaemia			
		Steady state *n* = 71	Painful crisis *n* = 71		

Low	46.7 (37.8)	29.3 (10.8)	20.0 (9.9)	1.9	.192
Moderate	40.6 (12.5)	37.6 (13.0)	25.7 (10.9)	23.1	.000
High	46.9 (10.8)	55.4 (11.5)	34.4 (11.5)	6.6	.004

## References

[1] Hendrickse RG, Hendrickse RG, Barr DCG, Matthews TS (1991). Disorders of the blood. *Paediatrics in the Tropics*.

[2] Serjeant GR, Serjeant GR (1988). Nomenclature and genetics of sickle cell disease, historical aspects. *Sickle Cell Disease*.

[3] Brewer GJ, Oelshlegel FJ (1974). Antisickling effects of zinc. *Biochemical and Biophysical Research Communications*.

[4] Adelekan DA, Thurnham DI, Adekile AD (1989). Reduced antioxidant capacity in paediatric patients with homozygous sickle cell disease. *European Journal of Clinical Nutrition*.

[5] Chow CK (1979). Nutritional influence on cellular antioxidant defense systems. *American Journal of Clinical Nutrition*.

[6] Hasanato RMW (2006). Zinc and antioxidant vitamin deficiency in patients with severe sickle cell anemia. *Annals of Saudi Medicine*.

[7] Leonard MB, Zemel BS, Kawchak DA, Ohene-Frempong K, Stallings VA (1998). Plasma zinc status, growth, and maturation in children with sickle cell disease. *Journal of Pediatrics*.

[8] Zemel BS, Kawchak DA, Fung EB, Ohene-Frempong K, Stallings VA (2002). Effect of zinc supplementation on growth and body composition in children with sickle cell disease. *American Journal of Clinical Nutrition*.

[9] Pories WJ, Henzel JH, Rob CG, Strain WH (1967). Acceleration of healing with zinc sulfate. *Annals of Surgery*.

[10] Prasad AS, Beck FWJ, Kaplan J (1999). Effect of zinc supplementation on incidence of infections and hospital admissions in sickle cell disease (SCD). *American Journal of Hematology*.

[11] Prasad AS, Abbasi AA, Rabbani P, DuMouchelle E (1981). Effect of zinc supplementation on serum testosterone level in adult male sickle cell anemia subjects. *American Journal of Hematology*.

[12] Warth JA, Prasad AS, Zwas F, Frank RN (1981). Abnormal dark adaptation in sickle cell anemia. *Journal of Laboratory and Clinical Medicine*.

[13] Prasad AS, Cossack ZT (1984). Zinc supplementation and growth in sickle cell disease. *Annals of Internal Medicine*.

[14] Gupta VL, Chaubey BS (1995). Efficacy of zinc therapy in prevention of crisis in sickle cell anemia: a double—blind, randomized controlled clinical trial. *The Journal of the Association of Physicians of India*.

[15] Prasad AS, Ortega J, Brewer GJ, Oberleas D, Schoomaker EB (1976). Trace elements in sickle cell disease. *Journal of the American Medical Association*.

[16] Akenami FO, Aken’Ova YA, Osifo BO (1999). Serum zinc, copper and magnesium in sickle cell disease at Ibadan, south western Nigeria. *African Journal of Medical Sciences*.

[17] Kirkwood BR, Kirkwood BR (1995). Calculation of required sample size. *Medical Statistics*.

[18] Familusi JB, Adeyokunnu AA (1990). Geographical distribution and pathogensis of the haemoglobinopathies. *DOKITA*.

[19] Ogunrinde GO, Yakubu AM, Akinyanju OO (2000). Anthropometric measures and zinc status of children with sickle cell anaemia. *Nigerian Journal of Paediatrics*.

[20] Akinkugbe OO, Akinkugbe OO (1992). Sickle cell disease. *Non Communicable Disease in Nigeria*.

[21] Juwah AI, Nlemadim EU, Kaine W (2004). Types of anaemic crises in paediatric patients with sickle cell anaemia seen in Enugu, Nigeria. *Archives of Disease in Childhood*.

[22] Smith JC, Butrimovitz GP, Purdy WC (1979). Direct measurement of zinc in plasma by atomic absorption spectroscopy. *Clinical Chemistry*.

[23] Whitehouse RC, Prasad AS, Rabbani PI, Cossack ZT (1982). Zinc in plasma, neutrophils, lymphocytes, and erythrocytes as determined by flameless atomic absorption spectrophotometry. *Clinical Chemistry*.

[24] Prasad AS, Oberleas D, Halsted JA (1965). Determination of zinc in biological fluids by atomic absorption spectrophotometry in normal and cirrhotic subjects. *The Journal of Laboratory and Clinical Medicine*.

[25] Balch AP, Balch PA, Balch JF (2000). Minerals. *Prescription for Nutritional Healing*.

[26] Norma MJT, Pearson CJ, Searle PGE (1996). *Tropical Food Crops in Their Environment*.

[27] Bahijri SM (2001). Serum zinc in infants and preschool children in the Jeddah area: effect of diet and diarrhea in relation to growth. *Annals of Saudi Medicine*.

[28] Akinkugbe FM, Ette SI (1987). Role of zinc, copper, and ascorbic acid in some common clinical paediatric problems. *Journal of Tropical Pediatrics*.

[29] Ploysangam A, Falciglia GA, Brehm BJ (1997). Effect of marginal zinc deficiency on human growth and development. *Journal of Tropical Pediatrics*.

[30] Phebus CK, Maciak BJ, Gloninger MF, Paul HS (1988). Zinc status of children with sickle cell disease: relationship to poor growth. *American Journal of Hematology*.

[31] King JC, Shames DM, Woodhouse LR (2000). Zinc homeostasis in humans. *Journal of Nutrition*.

[32] Raghunath R, Tripathi RM, Khandekar RN, Nambi KSV (1997). Retention times of Pb, Cd, Cu and Zn in children’s blood. *The Science of the Total Environment*.

[33] Lau A, Chan L, Lee M (2009). Electrolytes, other minerals and trace elements. *Basic Skills in Interpreting Laboratory Data*.

[34] Brown KH, Peerson JM, Rivera J, Allen LH (2002). Effect of supplemental zinc on the growth and serum zinc concentrations of prepubertal children: a meta-analysis of randomized controlled trials1-3. *American Journal of Clinical Nutrition*.

